# Spatial complexity of carcass location influences vertebrate scavenger efficiency and species composition

**DOI:** 10.1038/s41598-017-10046-1

**Published:** 2017-08-31

**Authors:** Joshua B. Smith, Lauren J. Laatsch, James C. Beasley

**Affiliations:** 1 University of Georgia, Savannah River Ecology Lab, PO Box Drawer E, Aiken, SC 29802 USA; 2 University of Georgia, Savannah River Ecology Lab, Warnell School of Forestry and Natural Resources, PO Box Drawer E, Aiken, SC 29802 USA

## Abstract

Scavenging plays an important role in shaping communities through inter- and intra-specific interactions. Although vertebrate scavenger efficiency and species composition is likely influenced by the spatial complexity of environments, heterogeneity in carrion distribution has largely been disregarded in scavenging studies. We tested this hypothesis by experimentally placing juvenile bird carcasses on the ground and in nests in trees to simulate scenarios of nestling bird carrion availability. We used cameras to record scavengers removing carcasses and elapsed time to removal. Carrion placed on the ground was scavenged by a greater diversity of vertebrates and at > 2 times the rate of arboreal carcasses, suggesting arboreal carrion may represent an important resource to invertebrate scavengers, particularly in landscapes with efficient vertebrate scavenging communities. Nonetheless, six vertebrate species scavenged arboreal carcasses. Rat snakes (*Elaphe obsolete*), which exclusively scavenged from trees, and turkey vultures (*Cathartes aura*) were the primary scavengers of arboreal carrion, suggesting such resources are potentially an important pathway of nutrient acquisition for some volant and scansorial vertebrates. Our results highlight the intricacy of carrion-derived food web linkages, and how consideration of spatial complexity in carcass distribution (i.e., arboreal) may reveal important pathways of nutrient acquisition by invertebrate and vertebrate scavenging guilds.

## Introduction

Research suggests a substantial number of animals die from causes other than predation, such as disease, and their carcasses become temporary food resources for a diversity of scavengers^1, 2^. Consequently, scavenging is an important mechanism facilitating energy flow throughout food webs that encompasses a wide range of organisms across multiple kingdoms^3–5^. Although carcasses are used extensively by invertebrates and microbes^6^, vertebrates often consume a substantive proportion of available carcasses^7, 8^, which varies spatially and temporally as a function of biotic and abiotic factors including temperature, habitat, carcass size, and presence of humans^3, 9–12^. Additionally, scavenging plays an important role in shaping communities through inter- and intra-specific interactions because the presence of carrion provides a temporary resource over which vertebrates, invertebrates, and microbes compete^2, 8, 13, 14^. Recent studies have shed light on the ecological^4, 5, 13, 15^ and economic^16^ importance of vertebrate scavengers, yet we are only beginning to understand how decomposition and scavenging dynamics differ within three-dimensional space^9, 14^.

In particular, spatial heterogeneity in the distribution of carrion (e.g., below ground, arboreal, under leaf litter, terrestrial surface) has largely been disregarded in scavenging studies, yet likely has profound effects on carcass fate, persistence times, and scavenger interactions^9^. Indeed, previous research shows microsite differences (e.g. upland vs. lowland habitats, open fields vs. burrows) can affect competition for carrion, mammalian contact rates – which could increase disease transmission, and decomposition times^11, 17, 18^. Although carrion predominantly occurs on the surface in terrestrial environments^19^, subterranean (e.g., carcasses in burrows), subsurface (e.g., rodents or caching of prey by predators), and arboreal carcasses also represent potentially important pathways of carrion availability^19–22^. However, we are not aware of any research that has evaluated the fate of arboreal carrion as most scavenging studies have experimentally placed carcasses on the ground. Such alternative forms of carrion availability, despite their reduced availability compared to surface carrion, could represent important pathways of nutrient acquisition by scavenging guilds often outcompeted for carrion resources, especially for invertebrates in landscapes with high densities of vertebrate scavengers able to consume 90% or more of available surface carrion^7^. For example, access to arboreal carrion is limited to volant or scansorial species and thus nutrients associated with such carcasses are likely available to a restricted suite of scavengers (both vertebrates and invertebrates) compared to surface carrion, potentially resulting in reduced scavenging efficiency by vertebrates. Moreover, disregarding such environmental heterogeneity is likely to result in a simplified understanding of functional redundancy within the scavenging community (e.g. ref. 23), and fails to account for potential emerging scavengers in novel environments.

Furthermore, despite the abundance and global distribution of birds and the fact that nesting mortality rates may be high^20, 24^, few studies have investigated the fate of avian carrion^25–27^ and no studies to date have examined the fate of juvenile bird carcasses. There are several causes of nestling mortality including insufficient parental care^28^, siblicide^29^, starvation^29^, disease^30^, extreme weather events, and abandonment^20^. All of these causes of death create carrion resources for scavengers. Nestling carcasses may be left in the nest, such as in the case of abandonment, or pushed out of the nest onto the ground, as in cases of siblicide. Although birds likely comprise a substantial proportion of arboreal carrion, arboreal carcasses are not only limited to birds as some reptiles (e.g., snakes, lizards) and mammals (e.g., squirrels, raccoons – *Procyon lotor*) den or concentrate activities in trees.

Given the lack of knowledge regarding the fate of arboreal carcasses, coupled with the fact that other forms of spatial heterogeneity in carrion availability contribute to considerable variability in scavenging community dynamics and thus nutrient cycling^1, 9, 10^, further research on the fate of non-surface carrion is needed to better elucidate food web dynamics in terrestrial ecosystems. Furthermore, data on scavenging of nestling bird carcasses will allow for a more comprehensive understanding of the ecological consequences of bird declines or implications of die-offs in terms of energy flow and transfer of toxicants^31^. To address knowledge gaps regarding the fate of nestling bird carcasses, as well as potential differences in the composition of scavenging communities between arboreal and ground-based carrion, our goal in this study was to test the hypotheses that (1) arboreal carrion represents a potentially important source of nutrients for scavenging communities, but that (2) scavenging rates by vertebrate scavengers and (3) vertebrate scavenger guilds differ between arboreal and terrestrial carrion. We predicted arboreal-placed carcasses would persist longer and be utilized by a smaller suite of vertebrate scavengers, thus potentially serving as critical resources for invertebrates, and even some specialized vertebrates, especially in areas with highly efficient vertebrate scavenging communities. This research provides novel evidence of scavenging ecology in a previously undocumented environment and allows us to predict how carcass locations influence nutrient recycling in forested ecosystems.

## Results

We conducted 214 scavenging trials over two summers, 120 trials between May and July 2015 and 94 from June to July 2016. We discarded one quail carcass placed in a tree nest due to the carcass blowing out of nest <3 hrs after placement. Overall, 16.4% (n = 35) of carcasses were scavenged by vertebrates and 4.2% (n = 9) had an unknown fate (Table 1). Of 107 carcasses placed in trees, 11 (10.3%) were scavenged by vertebrates compared to 24 (22.6%) of 106 carcasses placed on the ground (Table 1). Overall 16 (15.1%) quail and 19 (17.8%) chicken carcasses were scavenged (Table 1).

**Table 1:** Fate of 210 nestling chicken and quail carcasses that were monitored by remote cameras based on scavenging trials conducted 29 May to 29 July 2015 and 30 June to 27 July 2016 in forested plots at the Savannah River Site, Aiken, SC, USA.

Type	Scavenged by vertebrate	Scavenged by invertebrates	Unknown	Total	% scavenged by vertebrates
Overall	35	169	9	213	16.4
Chicken ground	14	36	3	53	26.4
Chicken tree	5	49	0	54	9.3
Quail ground	10	39	4	53	18.9
Quail tree	6	45	2	53	11.3
Ground	24	75	7	106	22.6
Tree	11	94	2	107	10.3
Chicken	19	85	3	107	17.8
Quail	16	84	6	106	15.1

**Figure 1:**
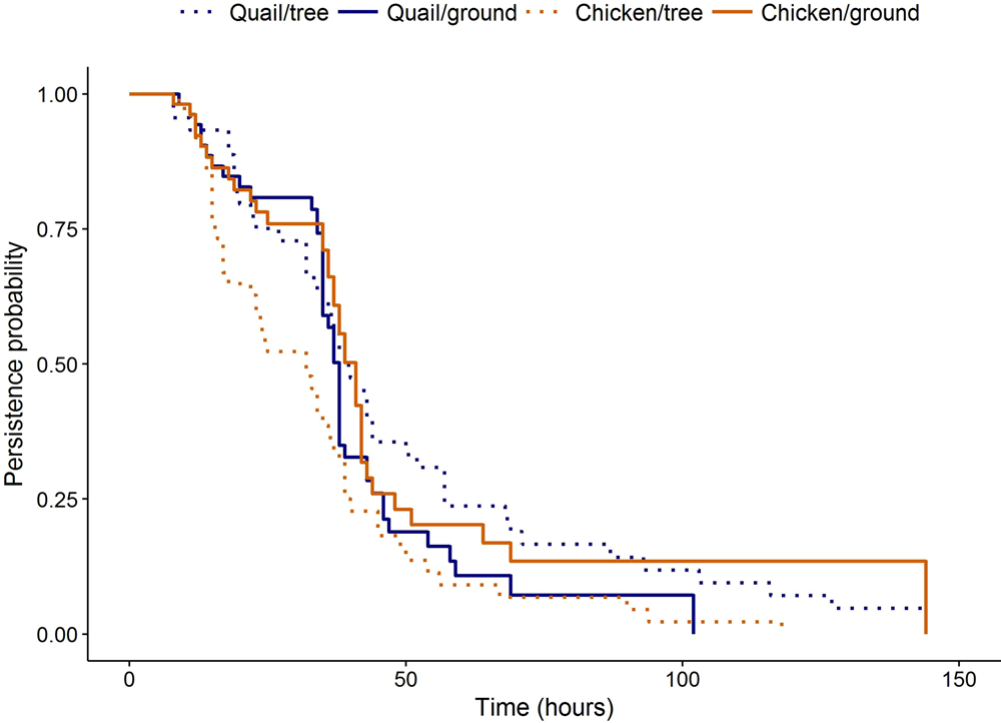
Probability of quail and chicken carcasses placed in trees and on the ground persisting on the landscape based on scavenging trials conducted 29 May to 29 July 2015 and 30 June to 27 July 2016 in forested plots at the Savannah River Site, Aiken, SC, USA. All carcasses scavenged by vertebrates were censored at time taken.

**Figure 2:**
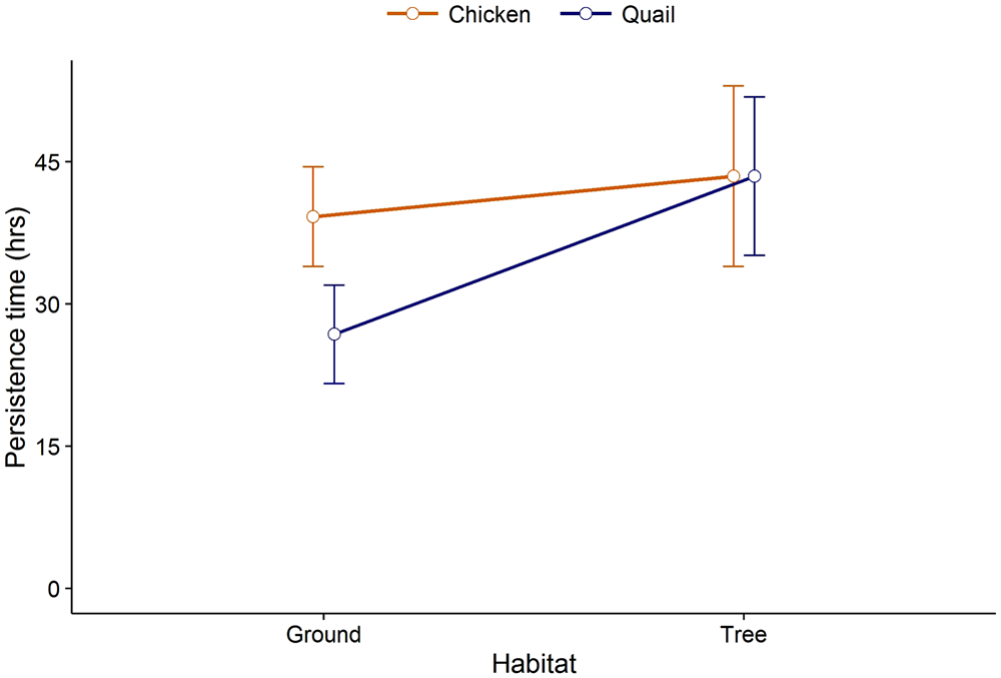
Average persistence time (i.e., decomposing or taken by invertebrates) with confidence intervals of quail and chicken carcasses placed in trees and on the ground (carcasses taken by vertebrates were censored at time of take) based on scavenging trials conducted 29 May to 29 July 2015 and 30 June to 27 July 2016 in forested plots at the Savannah River Site, Aiken, SC, USA.

Average persistence time was 38.7 hrs (SE = 2.0 hrs), although we observed a high degree of variability in decomposition time (Fig. 1). Carcasses placed in tree nests tended to remain available to potential scavengers longer than those on the ground, and in the absence of vertebrate scavengers, 50% of all carcasses were decomposed by invertebrates and microbes within 39 hrs (range 32 hrs for chicken carcasses placed in trees to 39 hrs for chicken placed on the ground and quail in trees; Fig. 1). Results of our log-rank test indicated significant differences (*X* ^2^_3_ = 10.3, *P* = 0.017) in persistence times between carcass sizes and habitat types. This was primarily due to quail carcasses placed on the ground persisting an average of 26.8 hrs (SE = 2.4 hrs) versus >42.3 hrs (SE = 2.4), on average, for quail placed in trees and for chicken carcasses in both habitat types (Fig. 2).

Among carcasses scavenged by vertebrates, overall average time until removal was 34.2 hrs (SE = 4.8 hrs), and ranged from 33.9 hrs (SE = 6.1 hrs) for those on the ground to 34.8 hrs (SE = 7.4 hrs) for carcasses in tree nests. Average time to removal of chicken carcasses was 44.0 hrs (SE = 7.3 hrs), compared to 22.5 hrs (SE = 4.5 hrs) for quail carcasses. Around the average removal times carcasses tended to be intact and exhibiting slight degradation from invertebrate activity. From the 35 positively identified vertebrate scavenger trials, model results constructed to determine differences in length of time until removal indicated the model containing only species was our best approximating model (*w*_*i*_ = 0.63). Remaining models were ≥2.0 ∆AIC*c* units from this model, and the weight of evidence supporting the top model was >1.7 times greater than all other models combined (see Supplementary Table S1). The β estimate for species was −0.690 (CI = −1.19 – −0.19) indicating quail carcasses were removed by vertebrates more quickly than chicken carcasses.

In contrast, linear mixed effect models constructed to examine difference in carcass fate (i.e., scavenged versus not scavenged by a vertebrate) indicated our most supported model was one that contained only habitat (see Supplementary Table S2). While one additional model fell within ≤2.0 ∆AIC*c* (model {habitat + species}), the β estimate for covariate *species* = 0 (CI = −0.97–0.54), thus, we considered this an uninformative variable^32^. The β estimate from our top model {habitat} was −1.15 (CI = −1.88 – −0.29) indicating carcasses placed in trees were less likely to be scavenged than those on the ground (Table S2).

Ten vertebrate species were documented scavenging ≥1 carcass during our trials, with two additional unknowns and one snake not identifiable to species. Of the 10 identifiable species, nine (90%) were observed scavenging ground-based carrion, while six (60%) were documented scavenging from trees (Table 2). Five species removed carcasses from both habitat types with rat snakes (*Elaphe obsolete*) representing the only species that scavenged exclusively from trees (Table 2). Overall, snakes were the most frequently observed scavenger taxa accounting for 14 (40.0%) of all scavenging events, and were represented by three different species; copperhead (*Agkistrodon contortrix*, n = 2), rat snake (n = 4), and black racer (*Coluber constrictor*, n = 7). Turkey vultures (*Cathartes aura*) accounted for eight (22.9%) scavenging events and raccoons (*Procyon lotor*) three (8.6%). Coyote (*Canis latrans*), gray fox (*Urocyon cinereoargenteus*), wild pigs (*Sus scrofa*), and Virginia opossum (*Didelphis virginana*) each accounted for two (5.7%) events, while an eastern box turtle (*Terrapene carolina*) was observed scavenging one carcass (2.8%; Table 2).

**Table 2:** Scavenger species take of experimentally-placed carcasses by habitat and carcass type based on scavenging trials conducted 29 May to 29 July 2015 and 30 June to 27 July 2016 in forested plots at the Savannah River Site, Aiken, SC, USA.

Scavenger species	Habitat		Carcass		total scavenged
	tree	ground	quail	chicken	
Turkey vulture	3	5	1	7	8
Raccoon	1	2	2	1	3
Wild pig	0	2	0	2	2
Opossum	1	1	0	2	2
Coyote	1	1	0	2	2
Fox	1	1	1	1	2
Black racer	0	7	4	3	7
Black rat snake	4	0	3	1	4
Copperhead	0	2	2	0	2
Snake sp.	0	1	1	0	1
Eastern box turtle	0	1	0	1	1
Unknown vertebrate	0	2	2	0	2
Total	11	25	16	20	36

**Figure 3:**
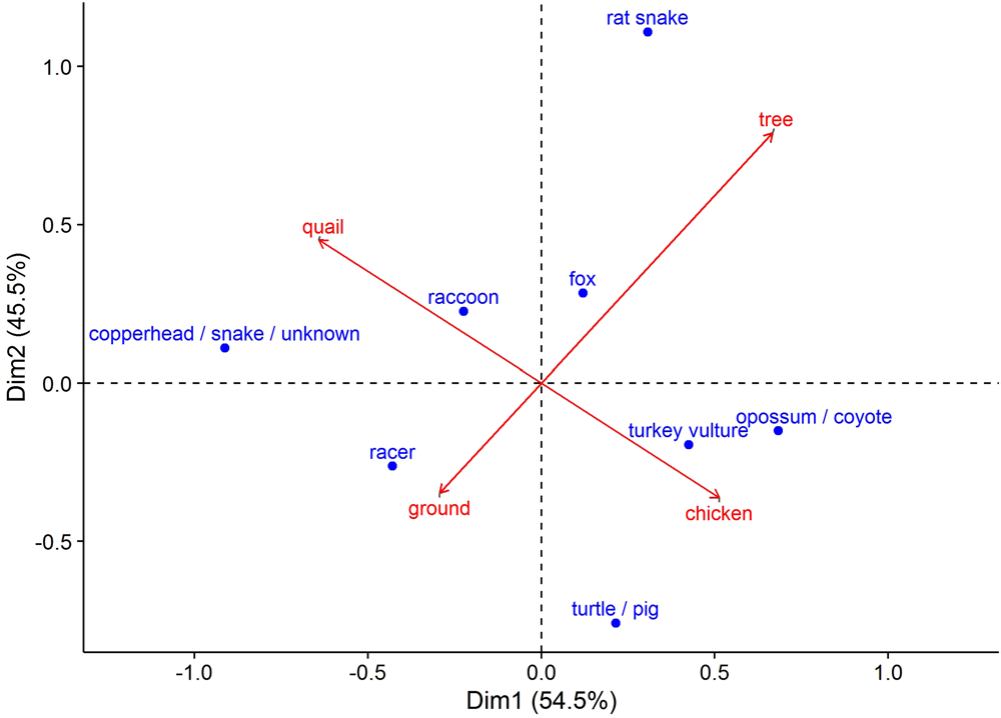
Figure shows scavenger species (blue dots), habitat and carcass type (red arrows) projected in top two major correspondence analysis dimensions with variance shown in parentheses based on scavenging trials conducted 29 May to 29 July 2015 and 30 June to 27 July 2016 in forested plots at the Savannah River Site, Aiken, SC, USA.

Percent occurrence of several scavenger species varied noticeably by habitat and carcass type (Fig. 3). Rat snakes exhibited the highest value along dimension 2, primarily due to all scavenging events for this species occurring in trees. In contrast, wild pigs and box turtles only scavenged ground carrion and exhibited the lowest value along dimension 2. Differences in dimension 1 were primarily driven by carcass type. The greatest dichotomy occurred between coyote/opossum and copperheads, due to their association with chicken and quail carcasses, respectively. Fox and raccoons were the least discriminatory for either carcass or habitat type, while black racer’s tended to take both carcass types from the ground.

## Discussion

Our study suggests arboreal carrion is potentially an important pathway of nutrient acquisition by some volant and scansorial vertebrates, especially rat snakes specialized in arboreal foraging. Nonetheless, terrestrial carcasses were scavenged at more than double the rate of arboreal carcasses and thus more energy is likely transferred from terrestrial carcasses to vertebrate scavengers than from arboreal carcasses. This suggests arboreal carrion may represent an important resource to invertebrate scavengers, particularly in landscapes where vertebrates consume nearly all available terrestrial carrion (e.g. ref. 7). The difference in scavenging rates observed among habitats was likely because terrestrial carcasses were available to a wider range of vertebrate scavenger species. In fact, of the ten vertebrate species we recorded scavenging, only rat snakes scavenged arboreal carcasses exclusively. The nests in our study were placed on relatively low branches of trees but had arboreal carcasses been higher up, the number of scavenged carcasses would likely have been lower. Several studies have shown mammalian predators more frequently depredate avian nests located lower to the ground, and nest success rates increase when nests are higher^33, 34^. In fact, Fisher and Wiebe^33^ found that, despite a difference of approximately 0.5 m, higher nests were more successful. Such small variations likely act within scavenging guilds as well, with arboreal carrion releasing some of the competitive pressures that exists for ground-based carrion. The added complexity of these environments could, however, expose new scavenger guilds in comparison to more competitive landscapes and function to reduce interspecific competition in comparison to less heterogeneous landscapes.

Carcasses that were not scavenged by vertebrates were unlikely to persist more than 48 hours, illustrating the efficiency at which invertebrates and microbes remove these items. Despite this relatively rapid removal, vertebrates managed to scavenge more than 16% of all carcasses, similar rates as those reported in the summer by DeVault *et al*.^1^ in 2004 –19% – for experimentally-placed mouse (*Mus musculus*) and rat (*Rattus norvegicus*) carcasses at our study site. Assuming that most pre-fledgling birds that die in the nest would be removed by parents or siblings and become terrestrial carrion, our results indicate nearly a quarter would likely be scavenged by vertebrates. The amount of biomass ultimately consumed by vertebrate scavengers would, however, likely depend on the size and/or age of the birds. For instance, our de facto young birds (i.e. quail carcasses) were removed by invertebrates at a significantly faster rate than our “older carcasses” (i.e. chicken carcasses), yet we found both large and small carcasses were scavenged by vertebrates at relatively equal proportions. Given the high percentage of nestling birds that die each year^20, 24^, even low levels of scavenging by vertebrates would represent an important pathway for energy transfer within these systems.

As a taxon, snakes were the dominant vertebrate scavengers, consuming 40% of scavenged carcasses, although this varied considerably by species. In general, snakes are uniquely suited to take advantage of the patchy and ephemeral nature of carrion. Their relatively low maintenance metabolism allows them to survive on a few feedings per year^35^, and their unique physical adaptations (e.g., no limbs, long slender body) would facilitate greater access to a variety of environments (e.g., burrows and tree canopies) less accessible to larger-bodied species. The physiological adaptations of snakes, as well as other ectotherms, would tend to indicate a substantial fitness advantage from scavenging, while their physical adaptations would allow them to occupy a niche that has traditionally been unassigned to other vertebrate taxon^36, 37^. Our results indicate at least some species of snakes (e.g., rat snakes) are ideally suited to access both living and dead biomass from arboreal environments. We also suspect snakes accounted for the majority of unknown carcasses that disappeared from view between time lapse photos. Although we placed carcasses on triggers, it was not uncommon for invertebrates to move them shortly after placement, and snake movement patterns were such that they rarely set off the motion sensor of cameras. Future research should utilize cameras and monitoring techniques that better detect snakes and other ectothermic organisms in order to determine the fates of all carcasses and minimize the amount of carcasses consumed by unknown scavengers.

Turkey vultures accounted for more scavenging events than any other species documented. Turkey vultures consumed a large proportion of arboreal carcasses in both years of our study, scavenging 24% (n = 5) of the scavenged carcasses in 2015 and 21% (n = 3) in 2016. This is in stark contrast to rates reported for previous studies investigating fate of small mammal (rats and mice) carrion, which have ranged from 0% to ~2% across several studies (e.g. refs 1, 7, 11 and 38). Although, within these same landscapes, Turner *et al*.^11^ and Olson *et al*.^39^ observed extensive scavenging of larger carrion by vultures, leading the authors to conclude that mammals were more efficient scavengers of small carcasses than vultures^11, 39^. The surprisingly high relative proportion of carcasses consumed by turkey vultures in our study is unlikely related to difference in mass compared to similar studies using small mammal carrion. For example, mouse (19.4 g; SD = 1.7) and rat (228 g; SD = 52) carcasses used by DeVault *et al*.^1^ were more than twice the mass of our quail and chicken carcasses, respectively. Of the two carcass types we deployed over the course of our study, we did observe that turkey vultures primarily consumed larger (i.e. chicken) carcasses (88%; Fig. 3), and three of the eight carcasses consumed (38%) were taken from arboreal nests. Turkey vultures are known for their keen sense of smell^40^. Consequently, it is possible the placement of carcasses even a few feet above ground aided in dispersing odors in such a way that they were more easily detected. Nevertheless, turkey vultures consumed similar proportions of carcasses from each habitat, removing 27% (n = 3) of arboreal carcasses that were scavenged by vertebrates and 21% (n = 5) of scavenged terrestrial carcasses. Thus, location and possible improved odor dispersal alone would not account for the 10 to 20 fold increase in scavenging rates observed between our study and others with similarly sized carcasses conducted on the SRS. The underlying mechanism contributing to differences among studies in turkey vulture scavenging of small carrion items is unknown, but could reflect increasing turkey vulture populations in the region^41^, possible differences in odor associated with avian versus mammalian carcasses or other unknown factors that warrant further investigation.

In addition to nestling birds, adult birds and other tree-dwelling species (e.g., squirrels, raccoons) die from causes other than predation (e.g. disease) and become carrion resources. Some of the carcasses will remain in trees (e.g. birds that die in cavities) while others may fall to the ground^22^. Scavenging is known to bring vertebrate species that do not typically interact into contact as they move into the same areas to compete over carrion resources^2^. Considering the number of arboreal species present in forested ecosystems, carrion originating from trees may be the basis of multiple links in food webs and connect arboreal, scansorial, and terrestrial vertebrates and could even connect arboreal species to burrowing species via invertebrates that move carrion from the trees to, and beneath, the forest floor. As ecosystems are continually affected by natural and anthropogenic stressors, understanding how scavenging guilds interact in heterogeneous environments to stabilize food webs, and ultimately influence ecosystem function, is increasingly important to understand. Our results highlight how even slight variation in carcass placement allows a spectrum of species to access carrion differentially. We recommend future researchers obtaining information on exact animal locations following mortality report these to allow for a more thorough assessment of the spatial variation of carrion availability, and inform additional empirical studies assessing its impact on scavenger efficiency.

## Methods

### Study Area

Our study was conducted at the Savannah River Site (SRS) near Aiken, South Carolina, USA. The SRS is a ~800 km^2^ site managed by the U.S. Department of Energy in the coastal plain region of South Carolina and borders the Savannah River. The SRS was closed to the public for nuclear production in the 1950s^42^. Prior to 1951, settlers cleared forests to build homesteads, for timber profit, and for conversion to agricultural land, specifically cotton and corn croplands^42^. A few areas remained unaltered, but after 1951 the USDA Forest Service restored much of the forest and continues to manage the majority of the SRS for timber production of pine^42^. Currently, 54% of the land on the SRS is managed pine for timber production, 21% is riparian corridors and wetland habitat, 11% is mixed forest, 9% is shrublands-grasslands, 3% is upland scrub forests, 2% is open water, and < 1% is developed for industrial purposes^43^. Industrial areas make up a small fraction of the overall land area of the SRS and thus the site represents important habitat for a diversity of wildlife species^42^.

### Field Methods

We selected two habitat types (ground vs. tree) and two carcass sizes (quail chicks and chicken chicks) to represent potential bird carrion size/age class and habitat scenarios found in nature. Frozen chicks of both species were ordered from an online store (RodentPro.com, LLC, Inglefield, IN) and a subset of carcasses were weighed; chicken carcasses ranged from 58.3–168.1 grams, with an average of 107.8 grams (SE = 3.5; n = 64) and quail carcasses weighed 4.8–11.1 grams, with an average of 8.1 grams (SE = 0.1; n = 82).

We conducted trials over two summers, 29 May to 29 July 2015 and 30 June to 27 July 2016, at nine mixed pine and hardwood stands separated by ≥1 km throughout the SRS. Our trials covered an area of ~170 km^2^ in the western portion of SRS. We selected sites based on access and distributed them over multiple forested plots to try and encompass a range of vertebrate species and densities. Trials were conducted using a randomized block design with the following possible trial types: 1) quail chick in a nest, 2) chicken chick on the ground, 3) quail chick on the ground, and 4) chicken chick in a nest. Trial type was determined by randomly selecting a condition for the first trial of each set of four and moving in numerical order to the next trial type, starting back at one after four.

Carcasses were monitored with Reconyx P9000 Hyperfire infrared, no-glow remote sensory cameras (RECONYX, Inc., Holmen, WI) equipped with a trigger system adapted from DeVault *et al*.^1^ that forced the camera to take a picture upon removal of a carcass from the switch. Cameras were programmed to take a set of three pictures when triggered by motion or the external trigger and wait 30 seconds before taking another set of images. Cameras were also programmed to take a time-lapse photo to monitor decomposition of carcasses between vertebrate scavenger visits, and in case vertebrates did not find the carcass before it was fully decomposed by invertebrates and microbes. In 2015 we set time lapse to 15-minute intervals, and adjusted this to 5-minute intervals in 2016 in an attempt to increase the probability of cameras capturing cold-blooded scavengers (i.e., snakes), as these species often would not trigger the motion sensor on the cameras. For terrestrial carcasses, we placed carcasses on the ground near the base of trees (both hardwood and pine) and under the tree’s canopy in order to mimic locations where nestlings may settle if removed from nests. We set trail cameras on a tree with the carcass in the center of the frame and camouflaged trigger cords with leaf litter. We placed arboreal carcasses in artificial nests (Factory Direct Craft Supply Inc., Springboro, OH) 105–173.5 cm (average = 133.6; SE = 1.7; n = 59) off the ground in trees with dense low branching. Thus, carcasses placed in tree nests were more indicative of low-canopy shrub-nesting species. We secured artificial nests with zip ties either on two branches or, if there were no branches sufficient to support the nest, nails were hammered into the tree and used as artificial branches. Nests were surrounded by other branches with leaves. We attached trail cameras monitoring arboreal nests to an adjacent tree positioned to look down onto the nest.

We placed a single carcass at each site and left carcasses in place for a minimum of six days before remains (if any) and the camera were recovered. Each active trial was at least 300 meters away from any other active trials and 100 meters away from any previous trial sites that year to ensure vertebrate scavengers did not become behaviorally conditioned to visit any specific site. We removed carcass remains (i.e. bones, feathers, flesh) at the end of the trial to prevent a lingering smell from attracting scavengers and biasing nearby trials. For trials that were in trees, we removed nests and nails at the conclusion of each trial.

### Camera Analysis

Images were downloaded from cameras after the conclusion of each trial and analyzed to determine: (1) the species, date, and time of all vertebrate scavenging events (i.e., vertebrate feeding on but not removing the entire carcass), (2) date and time each carcass was removed by a vertebrate scavenger, and (3) length of time carcasses that were not scavenged by a vertebrate persisted. A scavenging event was defined as any time a vertebrate consumed any part of the carcass. If we could not determine the exact species responsible for the scavenging event we classified them as unknown. Trial images were analyzed from the time the carcass was placed until a maximum of six days. Images were used to assess carcass fate (scavenged by vertebrates vs. decomposed by invertebrates), vertebrate scavenger species composition, and time to carcass removal. We did not attempt to quantify or categorize invertebrate species responsible for carcass decomposition as there were often multiple species present, and any sort of quantification would have been subjective. Rather, we used our time-lapse photos to determine that all carcasses not consumed by vertebrates were taken by invertebrates. All data generated or analyzed during this study are included in the Supplementary Information files.

### Statistical Methods

Differences in carcass fate, vertebrate scavenger community composition, and time to carcass removal were analyzed across habitat types and carcasses sizes. We used program R^44^ to perform all analyses. To assess differences in persistence time across habitat and carcass types, we used the survival package^45^ in program R to calculate probability of a carcass persisting through time for each group (e.g., chicken carcasses on the ground, and in trees). We considered carcasses no longer available when they were removed from sight of the camera (e.g., carried under leaf litter by invertebrates or out of camera view) or when it appeared invertebrates had removed all edible biomass. Time to removal was rounded to the nearest hour, and carcasses scavenged by vertebrates were censored at time taken. We used a log-rank test to evaluate whether observed differences in removal times varied by group. The test computes a χ^2^ for observed and expected events during each time step and tests the null hypothesis of no difference between curves.

We assessed time to removal for all carcasses taken by vertebrate scavengers using linear mixed effect models (LME) with the LME4 package^46^ in program R. Time to carcass removal was defined as the elapsed time between carcass placement and complete carcass removal by a vertebrate scavenger. We analyzed removal times as a function of species (i.e., size) and habitat type from all vertebrate-scavenged carcasses. Trials where no vertebrate scavenged the carcass were excluded from the analysis. We used time to removal (hrs) as the dependent variable, carcass and habitat types as independent variables, and included year and site (i.e., one of the nine mixed hardwood or pine stands) as random effects. We constructed five models to assess differences including a null model, both independent variables separately, and an additive and interactive effect of habitat and species. We used Akaike’s Information Criterion adjusted for small sample size (AIC_c_) to select models that best described the data. We compared AIC_c_ values to select the most parsimonious model and considered models differing by ≤2 AIC_c_ values as competitive^47^.

To evaluate differences in carcasses (1) scavenged vs (0) not scavenged by vertebrates, we used a generalized linear mixed effects model with the LME4 package^47^ in program R. We excluded all trials where we could not definitively determine if the carcass was scavenged by a vertebrate. We compared the five identical models used for detection times, and evaluated them using the same AIC_c_ framework.

To visualize the relationship between scavenger species and habitat and carcass types, we conducted a correspondence analysis (CA) using the R-package FactoMineR^48^. In our analysis, we used scavenger species as rows and habitat (tree or ground) and carcass type (quail or chicken) as columns. We then populated the table based on the number of scavenging events recorded for each scavenger species across all habitat type and carcass combinations. CA converts this frequency table data into a graphical display of the rows and columns and displays them as points, with distances between points corresponding to Chi-squared distances^49^. In a typical 2 dimensional CA plot, each axis reflects a certain amount of the total variance and points contributing most to the total variance fall furthest from the origin.

## Electronic supplementary material

Supplementary Table S1 and Supplementary Table S2

Dataset 1
