# Supplementary material for: Spatial complexity of carcass location influences vertebrate scavenger efficiency and species composition
Source: Sci Rep. 2017 Aug 31;7:10250. doi: 10.1038/s41598-017-10046-1 (PMC5578956; doi:10.1038/s41598-017-10046-1)
Supplement: Supplementary file 1 — Supplementary Table S1 and Supplementary Table S2 [file 41598_2017_10046_MOESM1_ESM.pdf]

**Supplementary Information.**

# Spatial complexity of carcass location influences vertebrate scavenger efficiency and species composition

Joshua B. Smith*, Lauren J. Laatsch[2], and James C. Beasley[2]

***University of Georgia, Savannah River Ecology Lab, PO Box Drawer E, Aiken, SC 29802, USA.

[2]University of Georgia, Savannah River Ecology Lab, Warnell School of Forestry and Natural Resources, PO Box Drawer E, Aiken, SC 29802, USA.

Correspondence and requests for materials should be addressed to J.S. (email: jbsmith1852@gmail.com)

**Supplementary Table S1.** Linear mixed effects models (LME) constructed to determine differences in length of time until carcass removal for nestling chicken and quail carcasses as a function of habitat and carcass type based on scavenging trials conducted 29 May to 29 July 2015 and 30 June to 27 July 2016 in forested plots at the Savannah River Site, Aiken, SC, USA.

| Model[a] | $K$[b] | AICc | Δ AICc[c] | $w$[d] | Cum.Wt | LL[e] |
|---|---|---|---|---|---|---|
| species | 5 | 94.18 | 0 | 0.63 | 0.63 | -41.05 |
| habitat+species | 6 | 96.71 | 2.53 | 0.18 | 0.81 | -40.85 |
| null | 4 | 97.73 | 3.55 | 0.11 | 0.92 | -44.2 |
| habitat*species | 7 | 99.21 | 5.03 | 0.05 | 0.97 | -40.53 |
| habitat | 5 | 100.33 | 6.16 | 0.03 | 1 | -44.13 |

[a]Habitat = carcass placed in a tree or on the ground, species = chicken or quail chicks. All models included site and year as random effects.

[b]Number of parameters.

[c]Difference in $AIC_c$ relative to min. $AIC_c$.

[d]Akaike wt.

[e]Log likelihood.

**Supplementary Table S2.** Linear mixed effects models (LME) constructed to determine differences in fate (scavenged vs not scavenged) of nestling chicken and quail carcasses as a function of habitat and carcass type based on scavenging trials conducted 29 May to 29 July 2015 and 30 June to 27 July 2016 in forested plots at the Savannah River Site, Aiken, SC, USA.

| Model[a] | $K^b$ | AICc | Δ AICc[c] | $w^d$ | Cum.Wt | LL[e] |
|---|---|---|---|---|---|---|
| habitat | 4 | 186.8 | 0 | 0.59 | 0.59 | -89.30 |
| habitat+species | 5 | 188.59 | 1.79 | 0.24 | 0.83 | -89.14 |
| habitat*species | 6 | 189.98 | 3.17 | 0.12 | 0.95 | -88.77 |
| null | 3 | 192.38 | 5.58 | 0.04 | 0.99 | -93.13 |
| species | 4 | 194.21 | 7.41 | 0.01 | 1 | -93.00 |

[a]Habitat = carcass placed in a tree or on the ground, species = chicken or quail chicks. All models included site and year as random effects.

[b]Number of parameters.

[c]Difference in $AIC_c$ relative to min. $AIC_c$.

[d]Akaike wt.

[e]Log likelihood.
